# Therapy Dogs' and Handlers' Behavior and Salivary Cortisol During Initial Visits in a Complex Medical Institution: A Pilot Study

**DOI:** 10.3389/fvets.2020.564201

**Published:** 2020-11-13

**Authors:** Stephanie D. Clark, Jessica M. Smidt, Brent A. Bauer

**Affiliations:** Division of General Internal Medicine, Section of Integrative Medicine and Health, Mayo Clinic, Rochester, MN, United States

**Keywords:** animal welfare, behavior, communication, cortisol, therapy dogs

## Abstract

Therapy dogs provide health benefits for individuals who suffer from illnesses, such as dementia, depression, loneliness, and aggression. Therapy dogs' impact on human health has been thoroughly studied; however, studies on dog welfare have been limited. Additionally, as dogs have evolved with humans, they have learned to read non-verbal social cues. Dogs can read humans' non-verbal body language and can react to their emotions. However, the body language of dogs is poorly understood and can lead to dog owner-directed aggression. Communication plays a vital role to be a cohesive therapy team. The purpose of this study was to assess perceived stress and cortisol concentrations in therapy dogs and their handlers during the first three visits in a hospital setting. Moreover, the study aimed to investigate whether, while in an overstimulating environment, a therapy dog handler can observe his or her dog's body language and correlate such observations to the dog's stress. Nine therapy dog teams from Mayo Clinic's Caring Canine Program participated in this study. A baseline salivary cortisol was collected from the handler and therapy dog each day of the visits. Once the team arrived, a pre-visit salivary cortisol was collected from the handler and therapy dog and, afterward, a post-visit salivary cortisol. Handlers were also asked to fill out a perceived stress survey on their own stress and that of their therapy dogs'. Behavior was documented by a staff member and the handler. For each visit, the therapy dogs were at the hospital on average 47 min and visited with nine people. There was significant correlation (*P* = 0.02) between the owner's perceived stress of his or her therapy dog and the dog's salivary cortisol concentrations. The handlers noted medium to high stress, and those dogs had higher cortisol concentrations post-visit. There was no significant difference in salivary cortisol for the handler and therapy dog over the course of the three visits and comparing pre- and post-visit. Overall, the dogs displayed mixed behaviors, with the three most reported being panting, lip licking, and yawning. However, salivary cortisol results suggest that the handlers and therapy dogs maintained their welfare state throughout the visits.

## Introduction

Dogs have been an integral part of humans' lives since the early Paleolithic time; however, the exact origin and date remain relatively vague (1). Not only have dogs created companions, but they have been noted to assist psychologically and emotionally. According to the Alliance of Therapy Dogs (2), as far back to the ancient Greeks, animals have been used to assist with mental and physical health. Since then, animals have been used for people who suffer from dementia, depression, loneliness, and aggression (2). It was not until the 1960s when the first research involving animal therapy was conducted by Boris Levinson (2). Since then, numerous research projects have been conducted investigating the positive benefits of therapy dogs for humans, but evaluating the animal's welfare during these sessions is limited.

Dogs have a variety of jobs to assist humans in their daily life and their health, such as service dogs, emotional support dogs, and therapy dogs. As humans and dogs have lived together, their bond has strengthened. Dogs have become better at observing human communication, verbal and non-verbal. Dog studies have observed that dogs can use non-verbal social cues from humans to achieve tasks, such as finding food (3, 4). Moreover, Kaminski et al. (5) studied facial expressions in humans. This study noted that human facial expressions are active communication attempts. Interestingly, it was observed that dogs can pick up on these small facial expressions and understand humans and react to the emotions (5).

On the other hand, it has been noted that dogs communicate mostly with their bodies and are trying to communicate with people multiple times a day (5). According to the American Society for the Prevention of Cruelty to Animals (ASPCA), there are key focal points other than overall posture and movement, such as ears, mouth, tail, hair, sweat, and ears that should be taken into consideration when reading a dog's body language (6). However, whether humans can read a dog's body language has been noted to be difficult especially for emotions, such as fear and anxiety (7). This leads to misinterpretation of what is being communicated and can lead to an escalation in emotions and actions. For example, fear and anxiety can escalate to aggression if the dog's body language is not understood (7).

Being able to read a family dog's body language is vital to avoid aggression especially toward children but is even more important for therapy dogs during therapy visits in a hospital setting. When therapy dogs are visiting in the hospital, there are various stimuli that can be stressful to the dogs. One study (8) stated that the therapy dog's handler's personality can also be a variation on how well the therapy team communicates. This study concluded that the handler's personality influences the team's performance; and agreeableness had the strongest correlation to the cooperation of the team, ability to avoid conflict, and reduced dog aggression toward the owner. Therefore, it is imperative that owners be able to accurately observe their dog's body language, listen to what their dogs are saying, and mitigate escalation of negative emotions.

Moreover, in addition to body language, cortisol has also been widely used in past literature (9–13) to assess animal welfare, especially as a non-invasive biomarker for stress in therapy dogs (14, 15). Cortisol is a hormone that aids in immune regulation and metabolism and involved in the body's stress response (16). Therefore, cortisol has been a preferred welfare biomarker to collect, especially in animal studies where behavior is observed (9–13, 17–23).

The purpose of this study was to assess perceived stress and cortisol concentrations in therapy dogs and their handlers during the first three visits in a hospital setting. Moreover, the study aimed to investigate if therapy dog handlers can observe their dog's body language while in an overstimulating environment and correlate their observations to their and their dog's salivary cortisol concentration.

## Materials and Methods

### Therapy Dog Teams

A therapy dog team consists of a dog and handler who have passed a therapy dog test and are considered a registered team. All teams that applied to the Caring Canines program at Mayo Clinic Rochester that had not volunteered with another dog or in another setting were asked to participate in the study. Handlers who had visited with other dogs were excluded, and dogs who had visited anywhere else were excluded as well. The team had to be a new team with no experience at Mayo Clinic, Rochester, MN, where all visits took place.

### Therapy Dogs

Nine therapy dog teams from Mayo Clinic, Rochester's Caring Canine Program, participated in this study. All dogs had passed a therapy dog examination and were healthy, up-to-date on vaccines, and not on a raw diet. The average age for the dogs was 3.7 SD ± 2.2 years. The demographics for the nine dogs were: Dog 1 was a 2-year-old male Soft Coated Wheaten Terrier registered with Pet Partners. Dog 2 was a 2.5-year-old male mixed breed registered with Alliance of Therapy Dogs. Dog 3 was a 4-year-old female Standard Poodle registered with Pet Partners. Dog 4 was a 3-year-old female mixed breed registered with Alliance of Therapy Dogs. Dog 5 was a 2-year-old male Labrador Retriever/Golden Retriever mix registered with Therapy Dogs International. Dog 6 was a 7-year-old female Australian Cattle Dog registered with Therapy Dogs International. Dog 7 was a 6-year-old male Golden Retriever registered with Alliance of Therapy Dogs. Dog 8 was a 7-year-old male Tibetan Terrier registered with Pet Partners. Dog 9 was 1.5-year-old male Standard Poodle registered with Therapy Dogs International. All dogs had a female handler except dog 9, which had a male handler. Mayo Clinic Rochester's IACUC committee approved this study (protocol A00003248-17).

**Figure 1:**
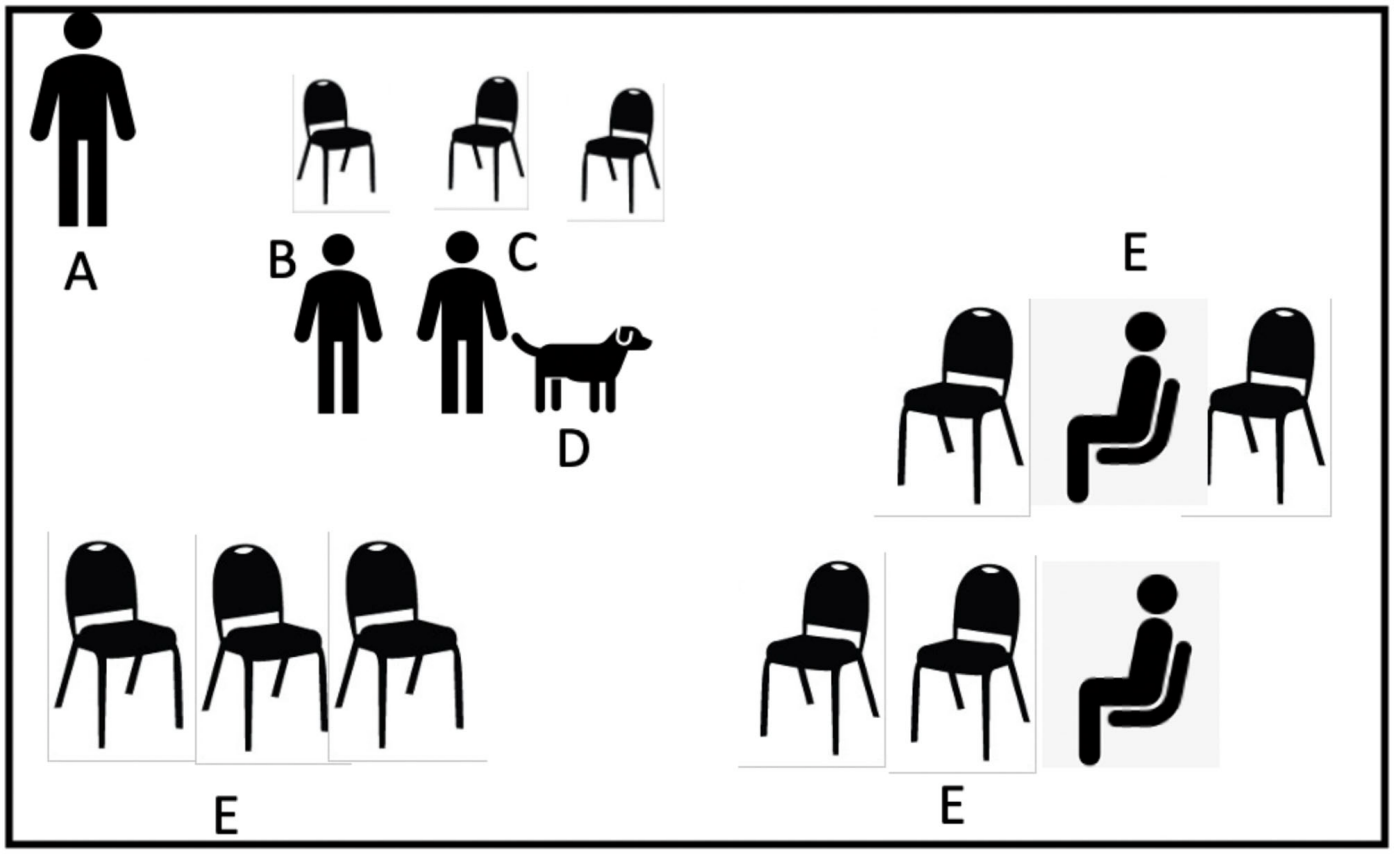
Visit 2, outpatient waiting room. A, study staff member; B, Caring Canine's volunteer coordinator; C, therapy dog handler; D, therapy dog; E, outpatient hospital waiting room, patients have the option to approach the therapy dog if patient wanted to.

**Figure 2:**
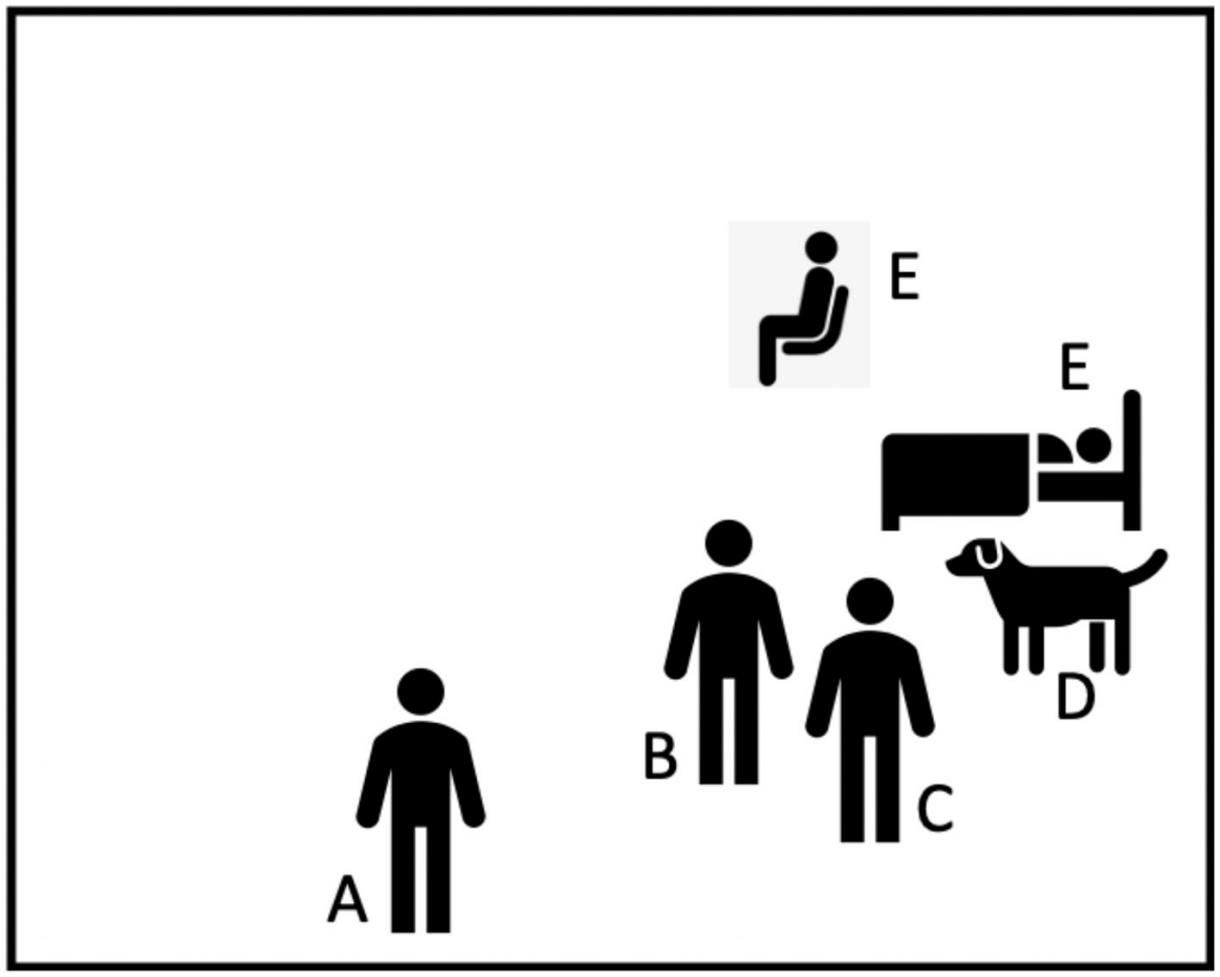
Visit 3, inpatient hospital rooms. A, study staff member; B, Caring Canine's volunteer coordinator; C, therapy dog handler; D, therapy dog; E, inpatient hospital (could be in hospital bed or in a hospital chair).

Before the start of the study, the handlers were asked to fill out general information about their dog and training techniques. During this time, the handlers were provided with a detailed description of each behavior that was written by a Karen Pryor Academy certified trainer. Each therapy team, when onboarded, had set three visits with the volunteer coordinator to adjust to the therapy dog visits. Visits consisted of inpatient and outpatient scenarios and were kept under an hour. Therapy dog visit duration, area of the hospital, and patient contact were recorded. The protocol followed the Mayo Clinic's Caring Canines protocol, including the first visit, walking around the hospital and getting accustomed to the layout and stimuli; the second visit was conducted in an outpatient waiting room; and the third visit was conducted in hospital rooms with patients. The first visit is used to get to know the campus, dogs could be touched and greeted but only in the hallways, and interactions were under 30 s. The second visit in the waiting room consisted of identifying a place to sit in the waiting room, and those interested in touching the dog could approach (Figure 1). The third visit was inpatient visits. The dog and handler would enter a hospital room where the patient would be either in his or her bed or sitting in the hospital chair (Figure 2). The dog could approach the patient and interact organically as this is how animal-assisted activities are conducted at Mayo Clinic. The team was accompanied by a study staff member to assist with saliva collection, surveys, and recorded behaviors they observed; this individual was only allowed to interact for collecting measurements. Additionally, a volunteer program leader whose role is to conduct the three sessions was present. This individual walks with the handler to direct the sessions. Only one therapy dog team was present for the visits. All therapy dog teams abided by the therapy dog policies of remaining on a 6-foot leash. Since not all therapy dog organizations allow giving treats during visits, handlers were refrained from rewarding dogs with treats for these visits. Dogs were allowed fresh water under the handler's direction. Mayo Clinic Rochester's Institutional Review Board committee approved this portion of the study (protocol 17-009412 00).

Throughout the duration of the study, the therapy dogs were not allowed to make any additional hospital visits or visit outside facilities. The day of each visit, the handler and therapy dog had saliva collected in the morning after the cortisol awakening response, before the visit, and directly after the meeting. Handlers were also asked to fill out a post-visit survey on how stressed they felt and the perceived stress of their dog. Handlers were also asked to list any behaviors noticed during the visit.

### Salivary Cortisol Analysis

Salivary cortisol was used as a non-invasive procedure to assess the stress response during the visit. The morning of each visit, 30 min after the therapy dog had woken up (24), a baseline saliva sample was collected using SalivaBio's Children's Swabs, Salimetrics LLC, Carlsbad, CA (25), by the dog's handler between 0600 and 0800 h. To remain consistent, while limited to the handler's availability, all handlers were asked to schedule each session between 1,100 and 1,500 h. Another saliva sample was collected before the start of the volunteering session (pre-visit), once the dog arrived at the campus. After the visit, on average 5 min after their last interaction, a post-visit sample was collected using the same technique for saliva collection at baseline. The delay in post-visit sampling was due to walking back to an exam room for collection. Each salivary collection was under 2 min to ensure the sampling did not affect the cortisol concentration (24). A swab method was used for the dogs as passive drool was not possible for these collections. The saliva swab was placed in a swab storage tube (SST) 17 × 100 mm (25). Handler saliva samples were also collected at the same frequency of the therapy dogs; however, this was collected via passive drool. All saliva samples in the storage tubes were stored in an 80°F until shipped on dry ice overnight to Salimetrics LLC, Carlsbad, CA, to be analyzed for salivary cortisol concentrations. Salimetrics cortisol assay has a sensitivity of <0.007 μg/dl. Additionally, individual percent coefficients of variance (CV) were calculated between the duplicates for each human and dog at each time point, and the averages of the percent CV were used to calculate the intra variance CV. Human intra-assay percent CV was 4.5% and dog was 5.4%. No inter-assay CVs were calculated due to samples not being measured over different plates or days.

### Surveys

A general introduction survey was administered prior to the start of the first visit. This survey contained questions regarding information on the therapy dog (i.e., breed, sex, and age), training techniques, and any training courses that may have been taken before testing to become a therapy dog. After every visit, the handler was given a modified perceived stress scale (26), which measures self-reported perceived stress and comfort level during the visits, with 0 being never/not stressed at all and 4 being very often/extremely stressed. At the end of this questionnaire, handlers were asked to rate their dog's perceived level of stress (low, medium, or high) and check off behaviors observed. Observed behaviors were combined with the study staff's observed behaviors to better capture the therapy dog's body language.

### Statistical Methods

For this pilot study, the average cortisol concentrations for visit (first, second, and third) and time of day (baseline, pre-session, and post-session) for dog and human samples were reported using least square means and standard errors. The perceived survey was a Likert scale questionnaire. Likert scale questions were described using frequency percentages and analyzed pairwise for each day combination using the Wilcoxon signed rank test for repeated measures.

Separate models were used to determine if the visit or time of day had any impact on the cortisol levels for the dogs or their handlers. Each of the nine pairs of participants had their cortisol checked three times a day on each visit, resulting in a sample size of 81 cortisol results. To account for fixed and random effects in the repeated measure design, linear mixed models with an outcome of cortisol concentrations were formulated for both dogs and handlers. The time of day that the cortisol sample was tested and the visit number were included as independent variables in the model. The ID of the dog/handler was included as a random effect in the model to control for the correlation between repeated measures. Akaike and Bayesian information criteria were used to compare covariance structures. For the linear mixed models for both handler and dog, a first-order autoregressive covariance structure was utilized.

To analyze the observed stress of the dogs from their handlers to the cortisol concentrations, generalized estimating equation (GEE) models were used to evaluate differences between visits using an outcome of low perceived stress vs. medium or high perceived stress. There was only one instance of high perceived stress, so the medium and high perceived stress answers were combined into one group. Perceived stress for each dog was collected on all 3 days for a total sample size of 27. The handler ID was included in the model as an identity variable. The independent variables of the cortisol level of the dog at the post-session time point and the visit day are included in the model. A *P* < 0.05 was considered significant in all cases. SAS (SAS version 9.4, SAS Institute Inc.) was used to analyze the data.

## Results

### Salivary Cortisol

The therapy dogs did not have any statistical significance when comparing salivary cortisol across time of day and between visits (Table 1). Baseline mean salivary cortisol concentrations were 0.59 SE ± 0.24, 0.32 SE ± 0.24, and 0.10 SE ± 0.23 for visits 1, 2, and 3, respectively. Pre-visit mean salivary cortisol concentrations were 0.07 SE ± 0.24, 0.17 SE ± 0.24, and 0.54 SE ± 0.26 for visits 1, 2, and 3, respectively. Lastly, post-visit mean salivary cortisol concentrations were 0.18 SE ± 0.23, 0.32 SE ± 0.23, and 0.20 SE ± 0.24 for visits 1, 2, and 3, respectively.

**Table 1:** Linear mixed models for comparison of cortisol concentrations between handlers and dogs over time.

	**Estimate (SE)**	***F*-value**	*P*-value^*^
**Dog model**			
Intercept	0.17		
Time of day		0.20	0.824
Baseline vs. Post	0.07 (0.16)		
Pre vs. Post	−0.02 (0.15)		
Visit		0.26	0.773
Day 1 vs. Day 3	0.12 (0.24)		
Day 2 vs. Day 3	0.15 (0.21)		
**Human model**			
Intercept	0.19		
Time of day		9.96	0.002
Baseline vs. Post	0.17 (0.04)		
Pre vs. Post	0.02 (0.04)		
Visit		0.38	0.687
Day 1 vs. Day 3	−0.05 (0.06)		
Day 2 vs. Day 3	−0.03 (0.05)		

* *ar(1) covariance structure*.

There were statistical differences (*P* = 0.002) in salivary cortisol concentrations across time of day for the handlers, with baseline being the highest concentration of salivary cortisol and post-session being the lowest (Table 1). Baseline mean salivary cortisol concentrations were 0.30 SE ± 0.06, 0.33 SE ± 0.06, and 0.38 SE ± 0.06 for visits 1, 2, and 3, respectively. Pre-session mean salivary cortisol concentrations were 0.17 SE ± 0.06, 0.17 SE ± 0.06, and 0.23 SE ± 0.06 for visits 1, 2, and 3, respectively. Lastly, post-session mean salivary cortisol concentrations were 0.16 SE ± 0.06, 0.18 SE ± 0.06, and 0.16 SE ± 0.06 for visits 1, 2, and 3, respectively.

### Survey Responses

There was significant correlation (*P* = 0.02) between the owner's perceived stress of their therapy dog and the dog's salivary cortisol concentrations (Table 2). The dogs' salivary cortisol concentration increased by 0.5 nmol/ml as the owner reported his or her dog experiencing medium or high stress compared to those who reported his or her dog to be low stressed during the visits.

**Table 2:** GEE model with low or medium/high perceived stress as the outcome.

	Odds Ratio (95% CI)^*^	***P*-value**
Visit 1	1.12 (0.14, 9.11)	0.914
Visit 2	0.44 (0.05, 3.83)	0.460
Visit 3	ref	ref
Cortisol concentration (unit = 0.5)^†^	0.18 (0.04, 0.79)	0.024

* Model outcome is the odds of handler perceiving low stress rather than medium or high stress.

† *Cortisol concentration at the post point for dog*.

There were no statistical differences in the perceived stress questions. However, when asked “During the visit, how often were you upset because something happened unexpectedly?” (Supplementary Table 1), nearly two-thirds of the handlers responded that they had never felt this way. The handlers (55.6%) responded with sometimes when asked how often they felt nervous or stressed, but the amount decreased throughout visits 2 and 3, with 11.1% responding with sometimes during each visit.

When asked “How often did you feel unable to control your dog?,” the handlers felt less confident as the visits progressed, with 66.7% responding with never during the first visit, 55.6% during the second, and 44.4% during the third visit. One handler responded that he or she had felt he or she did not have control over the dog nearly the entire time, but this was only for the first visit. Moreover, as the visits progressed, 55.6% of the owners responded almost never when asked how often they had to stop the visit because their dog was not comfortable. This was from visit 1 (22.2%) and visit 2 (11.1%). Lastly, the handlers reported that they had noticed their dog showing signs of stress sometimes during visit 1 (44.4%), 2 (55.6%), and 3 (33.3%).

### Observed Behavior

Over the course of the three visits, the therapy dogs were on the hospital campus for an average of 47 min (range: 25–60 min) and visited with nine people (range: 4–24 people) (Supplementary Table 2). Overall, the dogs displayed a mix of behaviors with the most common (22 out of the 27 visits) being panting. The second and third most frequent behaviors observed were lip licks (19) and yawning (14). Moreover, leaning into people the therapy dog was interacting with was noted in 13 out of 27 visits. Head turning away from stimulus (10) and “wet dog” shake (9) were additional behaviors observed during the visits.

## Discussion

### Salivary Cortisol

Salivary cortisol is used as a non-invasive measurement for assessing stress in therapy dogs (9–13). Baseline was set in correspondence with the cortisol awakening response, where the cortisol concentrations are at its peak (10). The cortisol awakening response is known to be one of the highest, naturally occurring cortisol peaks during the day due to the circadian rhythm, which provides a kick start to the production of cortisol within the first hour of waking up (10). The authors decided to use this as the baseline to determine if pre- or post-visit concentrations would exceed their natural peak. This was observed in this study's results. The baseline for all three visits had the largest cortisol concentration for the handlers and their dogs. While the pre- and post-visit cortisol concentrations were not significantly different, the post-visit concentrations were typically lower. This suggests that the handlers and dogs were not negatively affected by the visits (9–12). While previous studies have noted that handler's cortisol concentrations increased with the duration of the visit and the therapy dog's cortisol increased with the number of times they visited (10), this was not observed in the current study.

One limitation to this study is that salivary cortisol cannot be used individually for a definitive assessment of overall welfare, especially stress. However, this does give researchers a basis to further expand studies with additional parameters, such as heart rate variability, oxytocin concentrations, tympanic membrane temperature, and behavior of the dog (27–31). Moreover, handler compliance was assumed for participation during the study. The study staff explained and demonstrated the salivary collection procedure and provided details as to when to collect it; however, a study member was not present in the handler's home during the baseline collection. A second limitation was these were real sessions with new therapy dog teams and times could not be controlled for when the visit took place. To minimize the effect of the circadian rhythm on the cortisol concentrations, the authors asked that all visits be conducted between 1,100 and 1,500 h.

### Survey Responses

Utilization of a perceived stress survey for handlers to evaluate emotional welfare during therapy dog visits in a hospital is a relatively new tool. Interestingly, the handlers reported for most of the questions they either felt comfortable during the visits or did not feel upset when something unexpected happened. This suggests that the required pre-therapy visit classes and preparation that must be completed prior to the three visits may be preparing the handlers appropriately. Furthermore, Haubenhofer and Kirchengast (10) reported that therapy dog handlers described emotions experienced during therapy dog sessions like those emotions experienced in their everyday life. The positive descriptors reported were “interesting,” “joyful,” and “power dispensing,” while the negative descriptors were “stressful,” “physically and emotionally encumbering,” and “straining” (10).

The handlers in this study reported feeling less in control of their therapy dog as the visits progressed. While this did not align with the salivary cortisol concentrations, it is something that should be studied further to determine if this correlates to the stress increasing with the duration of the visits, which was observed by Haubenhofer and Kirchengast (10). Moreover, therapy dogs are given ratings during their therapy test, of “predictable” or “complex,” which can be considered on future studies if this has any impact on how dogs handle stress. Lastly, the handlers reported noticing their therapy dog becoming stressed at some point during the visits more than a third of the time for all visits, which seems to be common during therapy dog visits, regardless if it has an overall negative effect on the therapy dog's welfare (9, 12). Further studies should evaluate the welfare of therapy dogs over time to determine if there is a long-term effect from sometimes stressful therapy visits.

### Observed Behavior

While behavior can be subjective at times, it has been used as a non-invasive tool for assessing stress and overall welfare of dogs (12, 32). Of the observed behavior, the therapy dogs displayed a multitude of behaviors, with the most observed being panting, lip licks, yawning, leaning into people, turning away from a stimulus, and “wet dog” shake. Similar behaviors were observed by Glenk et al. (12). In the 2014 study, the reported behaviors were lip licks, yawning, paw lifting, body shake, tail wagging, and panting. In previous studies, lip licks and yawning have been associated with social conflict situations in dogs (33), and panting and tail wagging were associated with chronic stress (34). Additionally, lip licks and wet dog shake were correlated with higher cortisol concentrations during therapy dog visits (12). However, research has noted that these behaviors may be less related to the overall stress of the dog and more closely related to the dog coping with the stress (17, 35). A limitation of the observed behavior is relying on handlers to properly identify behaviors. The authors tried to account for this by providing handlers with a detailed description of the behaviors. Moreover, due to behavior being subjective and mixed studies on if it is related to the stress being experienced or a way of managing stressful situations, future studies should focus on quantifying behaviors and connecting them to specific events during a therapy visit. Additionally, future studies should apply the use of objective coding software (12) to analyzing behavior to provide a more reliable measurement. This was not utilized in this study due to study funding.

### Conclusion

This pilot study's results suggest that the handlers and therapy dogs maintained their welfare state throughout the visits and throughout the process. The non-invasive parameters utilized in this study suggested that the handlers and therapy dogs may have even been in a better welfare state post-visit. Furthermore, the significance in the handlers' assessment of his or her dog's perceived stress in correlation with the increase in therapy dog salivary cortisol suggests that the handler in a therapy team is perceptive to the therapy dog's welfare. One of the major limitations of this study was that salivary cortisol was the only biomarker collected. Past literature has demonstrated that cortisol can increase due to exercise and general arousal (positive or negative) (13, 15, 23). It is recommended that future studies use cortisol and additional biomarkers, such as tympanic membrane temperature (36), heart rate variability (37), and salivary oxytocin (27, 38). Furthermore, future studies would be beneficial in observing different therapy dog programs and their therapy teams' perceived stress along with the addition of objective physiological parameters.

## Data Availability Statement

The raw data supporting the conclusions of this article will be made available by the authors, without undue reservation.

## Ethics Statement

The studies involving human participants were reviewed and approved by Mayo Clinic Institutional Review Board. The patients/participants provided their written informed consent to participate in this study. The animal study was reviewed and approved by Mayo Clinic Rochester's IACUC committee approved this study (protocol A00003248-17). Written informed consent was obtained from the owners for the participation of their animals in this study.

## Author Contributions

SC contributed to the experimental design and formal analysis. SC and JS contributed to the performance of the experiment and data curation. SC, JS, and BB contributed to writing the original draft. All authors edited and approved the final manuscript.

## Conflict of Interest

The authors declare that the research was conducted in the absence of any commercial or financial relationships that could be construed as a potential conflict of interest.
