# Supplementary material for: Therapy Dogs' and Handlers' Behavior and Salivary Cortisol During Initial Visits in a Complex Medical Institution: A Pilot Study
Source: Front Vet Sci. 2020 Nov 13;7:564201. doi: 10.3389/fvets.2020.564201 (PMC7691227; doi:10.3389/fvets.2020.564201)
Supplement: Supplementary file 1 [file Data_Sheet_1.docx]

Supplementary Material

# Supplementary Data

Supplemental Table 1. Perceived stress post-visit Likert scale questionnaire^*^

|  | 0  Never | 1  Almost Never | 2  Sometimes | 3  Fairly Often | 4  Very Often |
| --- | --- | --- | --- | --- | --- |
| How often were you upset because something happened unexpectedly? | *Number of Handlers With Response (%)* | | | | |
| Visit 1 | 7 (77.8%) | 1 (11.1%) | 0 (0%) | 1 (11.1%) | 0 (0%) |
| Visit 2 | 6 (66.7%) | 2 (22.2%) | 1 (11.1%) | 0 (0%) | 0 (0%) |
| Visit 3 | 6 (66.7%) | 3 (33.3%) | 0 (0%) | 0 (0%) | 0 (0%) |
| How often did you feel unable to control your dog? |  |  |  |  |  |
| Visit 1 | 6 (66.7%) | 2 (22.2%) | 0 (0%) | 0 (0%) | 1 (11.1%) |
| Visit 2 | 5 (55.6%) | 3 (33.3%) | 1 (11.1%) | 0 (0%) | 0 (0%) |
| Visit 3 | 4 (44.4%) | 5 (55.6%) | 0 (0%) | 0 (0%) | 0 (0%) |
| How often did you feel nervous or stressed? |  |  |  |  |  |
| Visit 1 | 2 (22.2%) | 2 (22.2%) | 5 (55.6%) | 0 (0%) | 0 (0%) |
| Visit 2 | 4 (44.4%) | 4 (44.4%) | 1 (11.1%) | 0 (0%) | 0 (0%) |
| Visit 3 | 3 (33.3%) | 5 (55.6%) | 1 (11.1%) | 0 (0%) | 0 (0%) |
| How often did you not feel confident about your ability to handle problems that arose? |  |  |  |  |  |
| Visit 1 | 6 (66.7%) | 1 (11.1%) | 2 (22.2%) | 0 (0%) | 0 (0%) |
| Visit 2 | 6 (66.7%) | 1 (11.1%) | 1 (11.1%) | 0 (0%) | 1 (11.1%) |
| Visit 3 | 7 (77.8%) | 2 (22.2%) | 0 (0%) | 0 (0%) | 0 (0%) |
| How often did you feel that things were not going well? |  |  |  |  |  |
| Visit 1 | 6 (66.7%) | 2 (22.2%) | 1 (11.1%) | 0 (0%) | 0 (0%) |
| Visit 2 | 8 (88.9%) | 0 (0%) | 1 (11.1%) | 0 (0%) | 0 (0%) |
| Visit 3 | 6 (66.7%) | 3 (33.3%) | 0 (0%) | 0 (0%) | 0 (0%) |
| How often did you feel that you could not cope with something during the visit? |  |  |  |  |  |
| Visit 1 | 6 (66.7%) | 2 (22.2%) | 0 (0%) | 0 (0%) | 1 (11.1%) |
| Visit 2 | 7 (77.8%) | 2 (22.2%) | 0 (0%) | 0 (0%) | 0 (0%) |
| Visit 3 | 6 (66.7%) | 3 (33.3%) | 0 (0%) | 0 (0%) | 0 (0%) |
| How often were you unable to control your emotions and feelings? |  |  |  |  |  |
| Visit 1 | 6 (66.7%) | 2 (22.2%) | 0 (0%) | 0 (0%) | (11.1%) |
| Visit 2 | 7 (77.8%) | 2 (22.2%) | 0 (0%) | 0 (0%) | 0 (0%) |
| Visit 3 | 8 (88.9%) | 1 (11.1%) | 0 (0%) | 0 (0%) | 0 (0%) |
| How often did you feel angry that things were happening that were outside of your control? |  |  |  |  |  |
| Visit 1 | 9 (100%) | 0 (0%) | 0 (0%) | 0 (0%) | 0 (0%) |
| Visit 2 | 9 (100%) | 0 (0%) | 0 (0%) | 0 (0%) | 0 (0%) |
| Visit 3 | 8 (88.9%) | 1 (11.1%) | 0 (0%) | 0 (0%) | 0 (0%) |
| How often did you have to stop a visit because you felt your dog was not comfortable? |  |  |  |  |  |
| Visit 1 | 6 (66.7%) | 2 (22.2%) | 0 (0%) | 0 (0%) | 1 (11.1%) |
| Visit 2 | 6 (66.7%) | 1 (11.1%) | 2 (22.2%) | 0 (0%) | 0 (0%) |
| Visit 3 | 4 (44.4%) | 5 (55.6%) | 0 (0%) | 0 (0%) | 0 (0%) |
| How often did you see your dog showing signs of stress? |  |  |  |  |  |
| Visit 1 | 2 (22.2%) | 1 (11.1%) | 4 (44.4%) | 1 (11.1%) | 1 (11.1%) |
| Visit 2 | 1 (11.1%) | 2 (22.2%) | 5 (55.6%) | 0 (0%) | 1 (11.1%) |
| Visit 3 | 4 (44.4%) | 1 (11.1%) | 3 (33.3%) | 1 (11.1%) | 0 (0%) |

*All visits within questions were compared pairwise by visit using the Wilcoxon signed rank test. None of them were statistically significant.

Supplemental Table 2. Summary of therapy dog visits and displayed behaviors

| **Dog** | **Visit** | **Duration of Visit (min)** | **Human Interaction** | **Behaviors Observed** |
| --- | --- | --- | --- | --- |
| 1 | 1 | 31 | 5 | Yawn, Turn head away from stimulus, Lip lick, Panting, Refused treats, Lick those they interacted with |
| 1 | 2 | 45 | 4 | Yawn, Hypersalivating, Lip lick, Panting, Paw lift, Whale eye, Muzzle nudge, Paw for attention, Lean into people, Lick those they interacted with, Whining |
| 1 | 3 | 45 | 15 | Yawn, Hypervigilant activity, Turn head away from stimulus, Lip lick, Panting, Rest quietly, Lick those they interacted with |
| 2 | 1 | 45 | 5 | Yawn, Hypervigilant activity, Lip lick, Hypersalivation, Panting, Paw lift, Muzzle nudge, Lean into people, Rest quietly, Lick those they interacted with, Whining |
| 2 | 2 | 60 | 10 | Yawn, Sneeze, Hypervigilant activity, Turn head away from stimulus, Lip lick, Hypersalivation, Panting, Paw lift, Pacing, Muzzle nudge, Pawing for attention, Lean into people, Rest quietly, Lick those they interacted with, Whining |
| 2 | 3 | 45 | 12 | Yawn, Sneeze, Hypervigilant activity, Lip lick, Hypersalivation, Panting, Paw lift, Pacing, Wet dog shake, Whale eye, Muzzle nudge, Paw for attention, Lean into people, Rest quietly, Lick those they interacted with |
| 3 | 1 | 50 | 7 | Tremble, Panting, Lip lick |
| 3 | 2 | 55 | 11 | Panting, Wet dog shake, Lip lick, Rest quietly |
| 3 | 3 | 52 | 11 | Panting, Lip lick, Rest quietly |
| 4 | 1 | 40 | 6 | Lean into people |
| 4 | 2 | 44 | 8 | Turn head away from stimulus, Panting, Lean into people, Rest quietly, Lick those they interact with |
| 4 | 3 | 25 | 4 | Panting, Wet dog shake, Muzzle nudge, Lean into people, Rest quietly, Lick those they interact with |
| 5 | 1 | 32 | 11 | Turn head away from stimulus, Wet dog shake, Hiding/Avoiding interaction, Rest quietly, Tail tucked |
| 5 | 2 | 60 | 10 | Turn head away from stimulus, Hiding/Avoiding interaction, Rest quietly, Lick those they interact with, Tail tucked |
| 5 | 3 | 60 | 19 | Panting, Wet dog shake, Hiding/Avoiding interactions, Rest quietly, Lick those they interact with, Tail tucked |
| 6 | 1 | 43 | 10 | Yawn, Sneeze, Turn head away from stimulus, Lip Lick, Panting, Lean into people, Lick those they interact with, Pulled out of leash, Showed teeth |
| 6 | 2 | 60 | 9 | Tremble, Panting, Refused treats, Wet dog shake, Lean into people, Rest quietly, Lick those they interact with, Showed teeth |
| 6 | 3 | 60 | 20 | Tremble, Panting, Refused treats, Wet dog shake, Lean into people, Lick those they interact with, Showed teeth |
| 7 | 1 | 60 | 5 | Yawn, Sneeze, Lip lick, Panting, Wet dog shake, Rest quietly, Lick those they interact with |
| 7 | 2 | 40 | 24 | Yawn, Turn head away from stimulus, Lip lick, Panting, Hypersalivation, Wet dog shake, Rest quietly Lean into people |
| 7 | 3 | 35 | 7 | Panting, Lip lick, Turn head away from stimulus, Lean into people |
| 8 | 1 | 47 | 10 | Yawn, Lip lick, Whale eye, Muzzle nudge, Lean into people, Rest quietly, Lay down |
| 8 | 2 | 45 | 5 | Panting, Hypervigilant activity, Lip lick, Wet dog shake, Tail tucked, Turn head away from stimulus, Hiding/Avoiding interactions |
| 8 | 3 | 44 | 5 | Yawn, Lip lick, Paw lift, Muzzle nudge, Paw for attention, Rest quietly |
| 9 | 1 | 45 | 2 | Yawn, Panting, Lip lick, Lick those they interact with |
| 9 | 2 | 60 | 14 | Yawn, Panting, Lip lick, Hiding/Avoiding interactions, Muzzle nudge, Paw for attention, Lean into people |
| 9 | 3 | 60 | 19 | Yawn, Panting, Lip lick, Hiding/Avoiding interactions, Muzzle nudge, Lean into people |

**
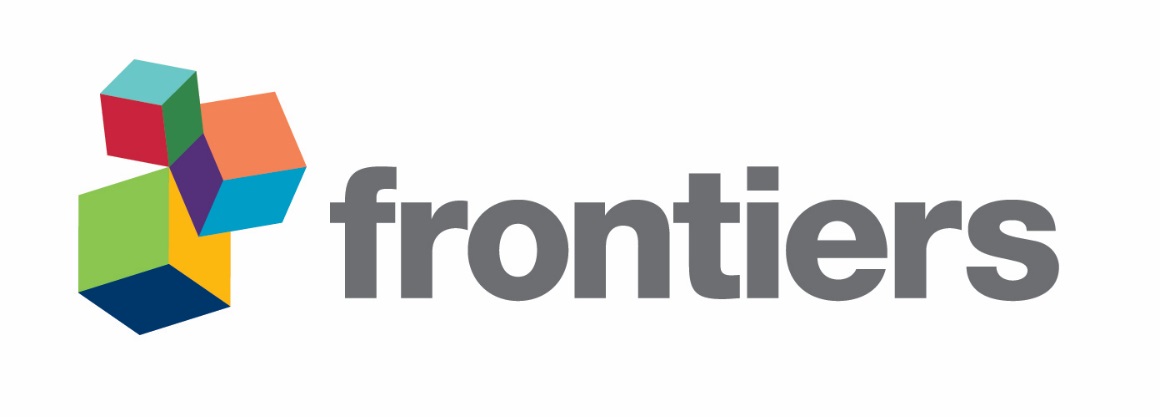
**
